# Accelerating scientific discovery with Co-Scientist

**Published:** 2026-06-29

**Authors:** Juraj Gottweis, Wei-Hung Weng, Alexander Daryin, Tao Tu, Petar Sirkovic, Artiom Myaskovsky, Grzegorz Glowaty, Felix Weissenberger, Alessio Orlandi, Dan Popovici, Anil Palepu, Keran Rong, Ryutaro Tanno, Khaled Saab, Fan Zhang, Jacob Blum, Andrew Carroll, Kavita Kulkarni, Nenad Tomašev, Dina Zverinski, Ivor Rendulic, Elahe Vedadi, Florian Hasler, Luka Rimanic, Marina Boia, Ivan Budiselic, Ben Feinstein, Mathias Bellaiche, Tom Sheffer, Jan Freyberg, Jeremy Ratcliff, Ottavia Bertolli, Katherine Chou, Avinatan Hassidim, Burak Gokturk, Amin Vahdat, Yuan Guan, Vikram Dhillon, Eeshit Dhaval Vaishnav, Byron Lee, Tiago R D Costa, José R Penadés, Gary Peltz, Yossi Matias, James Manyika, Demis Hassabis, Yunhan Xu, Pushmeet Kohli, Annalisa Pawlosky, Alan Karthikesalingam, Vivek Natarajan

**Affiliations:** 1 Google Cloud AI Research, Zurich, Switzerland; 2 Google DeepMind, Mountain View, California, USA; 3 Google Research, Mountain View, California, USA; 4 Stanford University School of Medicine, Palo Alto, California, USA; 5 Houston Methodist, Houston, Texas, USA; 6 Sequome, South San Francisco, California, USA; 7 Fleming Initiative and Imperial College London, London, UK

## Abstract

Scientific discovery is driven by scientists generating novel hypotheses for complex problems that undergo rigorous experimental validation. To augment this process, we introduce Co-Scientist, a multi-agent AI system built on Gemini for structured scientific thinking and hypothesis generation. Co-Scientist aims to help scientists discover new original knowledge. Conditioned on their research objectives and prior scientific evidence, it formulates demonstrably novel research hypotheses for experimental verification. The system’s design involves agents continuously generating, critiquing and refining hypotheses accelerated by scaling test-time compute. Key contributions include: (1) a multi-agent architecture with an asynchronous task execution framework for flexible compute scaling; (2) a tournament evolution process for self-improving hypotheses generation. Automated evaluations show continued benefits of test-time compute scaling, improving hypothesis quality over time. While general purpose, we focus the validation in three biomedical applications: drug repurposing, novel target discovery ^1^, and explaining mechanisms of anti-microbial resistance ^2^. Specifically, Co-Scientist helped identify new drug repurposing candidates and synergistic combination therapies for acute myeloid leukemia, which were validated through *in vitro* experiments. These real-world validations demonstrate the potential of Co-Scientist to accelerate scientific discovery and usher in an era of AI empowered scientists.

## Introduction

Researchers are faced with a breadth and depth conundrum. The complexity of scientific topics require increasingly deep and specific subject matter expertise, while leaps in insight may still arise from broad knowledge bridging across disciplines ^3–5^. With the rapid rise in scientific publications and the development of numerous specialized technologies, mastery of both discipline-specific depth and trans-disciplinary insight can be challenging.

At the same time, there has been rapid technological progress in AI towards generally intelligent and collaborative systems, which might empower scientists in creatively traversing and expertly reasoning across disciplinary domains. Such systems are capable of advanced reasoning ^6–8^, multimodal understanding ^8^, and agentic actions ^9^, such as the ability to use tools to solve complex tasks over long time horizons. Further, the trends with distillation ^10^, and inference time compute costs ^8,11^, indicate that such intelligent and general AI systems are rapidly becoming more accessible. Motivated by the aforementioned unmet needs in the modern discovery process in science and medicine and building on the advancements in frontier AI ^12^, we develop and introduce Co-Scientist.

**Fig. 1 |:**
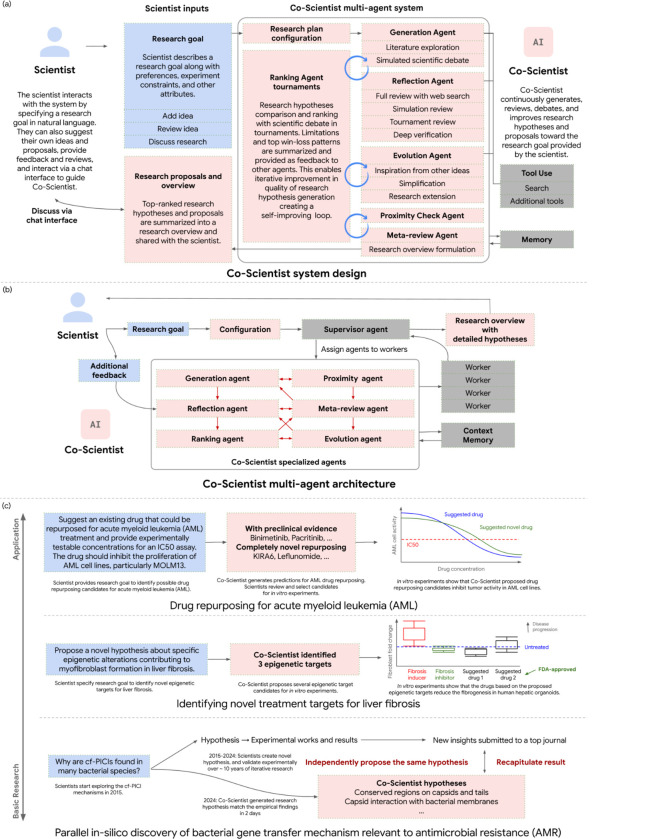
Co-Scientist design, multi-agent architecture, and experimental validation summary. (a) Overview: We illustrate the different components of Co-Scientist’s structured scientific thinking engine—the multi-agent system, and its interaction paradigm with scientists. Given a research goal in natural language, Co-Scientist generates novel research hypotheses. The system employs specialized Gemini based agents, including Generation, Reflection, Ranking, Evolution, Proximity (which evaluates relatedness), Meta-review (which provides high level analysis) agents, to continuously generate, debate, and evolve research hypotheses within a tournament framework. Feedback from the tournament enables iterative improvement, creating a self-improving loop towards novel and high-quality hypotheses for solving complex scientific problems. Co-Scientist leverages tools, including web search and specialized AI models to improve the grounding and quality of generated research hypotheses. Scientists can converse with Co-Scientist in natural language to specify research goals, incorporate constraints, provide feedback, steer and suggest new directions for explorations via the designated user interface. (b) The underlying multi-agent architecture: A Supervisor agent parses the user’s natural language research goal and dynamically allocates resources to specialized worker agents within an asynchronous task queue. The red boxes in the “Co-Scientist specialized agents'” section denote individual agents each with their own unique logic and role. The blue boxes indicate the scientist-in-the-loop inputs and feedback. The dark gray arrows represent the information flow through the Co-Scientist, while the red arrows represent the information feedback loop between the specialized agents. (c) End-to-end validation of Co-Scientist across three biomedical problems of varying complexity: Co-Scientist proposed novel drug repurposing candidates for acute myeloid leukemia (top), identified novel epigenetic targets for liver fibrosis (middle), and independently recapitulated a (then) unpublished, co-timed discovery of a novel bacterial gene transfer mechanism relevant to antimicrobial resistance (bottom). All Co-Scientist generated hypotheses were subsequently verified through independent *in vitro* laboratory experiments.

Co-Scientist is a structured scientific thinking engine designed to act as a collaborator to scientists and help accelerate the scientific discovery process. The system is a compound, multi-agent AI system ^13^ building on Google’s large language model (LLM), Gemini ^14^, mirroring the reasoning process underpinning the scientific method ^15^. Given a research goal specified in natural language, the system can search, learn and reason over relevant literature to synthesize prior work and propose novel, original research hypotheses and experimental protocols (Fig. 1a). Co-Scientist provides grounding for its recommendations by citing relevant literature, applying sound scientific reasoning and verifying its conclusions through external tools when applicable.

Co-Scientist is purpose-built for a “scientist-in-the-loop” collaborative paradigm. Scientists can specify their research goals in simple natural language and inform the system of desirable attributes and constraints for the proposed solutions. They can also actively interact with, and steer the system, including directly suggesting initial ideas and hypotheses for the exploration, refining generated ideas, or providing feedback through natural language chat.

Co-Scientist works through a significant scaling of the test-time compute paradigm ^16–18^ implementing structured scientific thinking in a multi-agent setup to iteratively reason, evolve, and improve the outputs as it gathers more knowledge (Fig. 1b). Underpinning the system are thinking and reasoning steps—notably a self-play based scientific debate step for generating novel research hypotheses; tournaments that compare and rank hypotheses via the process of finding win and loss patterns, and an evolution process to improve their quality. Finally, the agentic nature of the system enables it to recursively self-critique its output and use tools such as web-search and specialized AI models to provide itself with feedback to refine its hypotheses and research proposals.

While Co-Scientist is general purpose and applicable across scientific disciplines, we validate it in three impactful areas of biomedicine with varied complexity: drug repurposing for cancer, novel treatment target discovery for liver fibrosis, and new mechanistic explanations for antimicrobial resistance (Fig. 1c).

Drug development remains an expensive and protracted process, with most new approvals requiring de novo discovery for each indication ^19^. Systematic identification of new therapeutic indications for approved agents via drug repurposing offers a pragmatic strategy to accelerate development timelines and reduce attrition ^20^. Using Co-Scientist, we generated large-scale repurposing predictions validated through expert curation and *in vitro* assays. The system proposed several single-agent and combination therapies for AML that demonstrate selective cytotoxicity at clinically relevant concentrations. Beyond repurposing, Co-Scientist enables hypothesis generation for de novo target discovery, a process traditionally limited by the scale and uncertainty of biological inference. We applied Co-Scientist to liver fibrosis, where it proposed and ranked novel epigenetic targets demonstrating significant anti-fibrotic activity and hepatocyte regeneration in human hepatic organoids ^1^. Finally, we examined bacterial gene transfer mechanisms related to antimicrobial resistance (AMR), a system-level challenge involving molecular mechanisms and evolutionary pressures ^2^. Researchers instructed the system to explore a topic their group had independently discovered, but not yet published. Co-Scientist was asked to hypothesize how capsid-forming phage-inducible chromosomal islands (cf-PICIs) exist across bacterial species. It independently proposed that cf-PICIs interact with diverse phage tails to expand host range, mirroring the researchers’ unpublished experimental findings detailed in co-timed reports ^2,21^.

Overall, our key contributions are summarized as follows: (1) **Introducing Co-Scientist.** We develop and introduce Co-Scientist, a structured scientific thinking engine, that goes beyond literature summarization and “deep research” tools to assist scientists in uncovering new knowledge, novel hypothesis generation, discovering unexpected connections, and experimental planning. (2) **Significant scaling of the test-time compute paradigm for scientific reasoning.** Co-Scientist is built on a Gemini based multi-agent architecture, utilizing an asynchronous task execution framework. This framework allows the system to flexibly allocate computational resources to scientific reasoning, mirroring key aspects of the scientific method. Specifically, the system uses self-play strategies, including a scientific debate and a tournament-based evolution process, to iteratively refine hypotheses and research proposals creating a self-improving loop. Using automated evaluations across 15 complex expert curated open scientific goals, we demonstrate the benefits of scaling the test-time compute paradigm with Co-Scientist outperforming other state-of-the-art (SOTA) agentic and reasoning models in generating high quality hypotheses for complex problems. (3) **Expert-in-the-loop scientific workflow.** Our system is designed for collaboration with scientists. The system can flexibly incorporate conversational feedback in natural language from scientists and co-develop, evolve and refine outputs. (4) **End-to-end validation of Co-Scientist in important topics in biomedicine.** We present end-to-end validation of novel AI-generated hypotheses through new empirical findings in three distinct and increasingly complex areas of biomedicine: drug repurposing, novel target discovery, and antimicrobial resistance (Table 1, Supplementary Note 1).

### Co-Scientist overview

Given a research goal, Co-Scientist generates hypotheses constrained by default criteria including plausibility, novelty, testability, and safety. At a high level, it employs an asynchronous multi-agent architecture where a team of agents co-operate to solve scientific problems and develop novel hypotheses (Fig. 1b). It comprises a natural language interface for expert supervision, a task execution framework for resource allocation, a suite of specialized agents (Generation, Reflection, Ranking, Evolution, Proximity, Meta-review) mirroring the scientific method, and a persistent context memory for long-horizon reasoning. Detailed system configurations and agent mechanisms are fully described in the Methods.

### System analysis and evaluation

We first conduct the initial system evaluations to benchmark and verify the choice of the architecture and metrics underpinning Co-Scientist (detailed in Supplementary Note 2, Supplementary Fig. 1). We perform an ablation study to investigate the contribution of each agentic component in Co-Scientist. We then analyze the impact of scaling test-time compute, and undertake a small-scale evaluation with domain experts to assess the quality of the system outputs. Finally, to assess the practical utility of the system’s novel predictions, we perform end-to-end wet-lab validations (laboratory experiments) of Co-Scientist-generated hypotheses and research proposals in three key biomedical applications: drug repurposing, discovering novel treatment targets, and elucidating the mechanisms underlying antimicrobial resistance (Table 1, Supplementary Note 1). The varying complexity and nature of these applications enable a more comprehensive assessment of the system. Notably, all three validations involved expert-in-the-loop.

#### Agent ablation analysis.

Our ablation analyses, detailed in Methods, Supplementary Note 3, and Supplementary Fig. 2–6, confirmed the importance of our multi-agent architecture and specialized prompting strategies for robust scientific reasoning. For instance, granting the Reflection agent access to external search tools effectively prevented the hallucination of seemingly novel but implausible hypotheses, while employing a scientific debate prompt in the Ranking agent significantly improved the ranking of hypotheses and reduced positional bias. Furthermore, iterative refinement by the Evolution agent substantially boosted hypotheses quality.

#### Scaling test-time compute improves scientific reasoning.

To evaluate the effects of test-time compute scaling and Co-Scientist’s progress during iterative scientific reasoning and hypothesis generation, we measured the Elo ratings of Co-Scientist generated hypotheses and proposals over the course of its thinking and computation (i.e. the tournament of hypotheses). This analysis was done across 203 distinct research goals curated across broad scientific topics (predominantly in biomedicine, but also included other topics such as mathematics and physics) and entered into Co-Scientist until February 3, 2025.

**Fig. 2 |:**
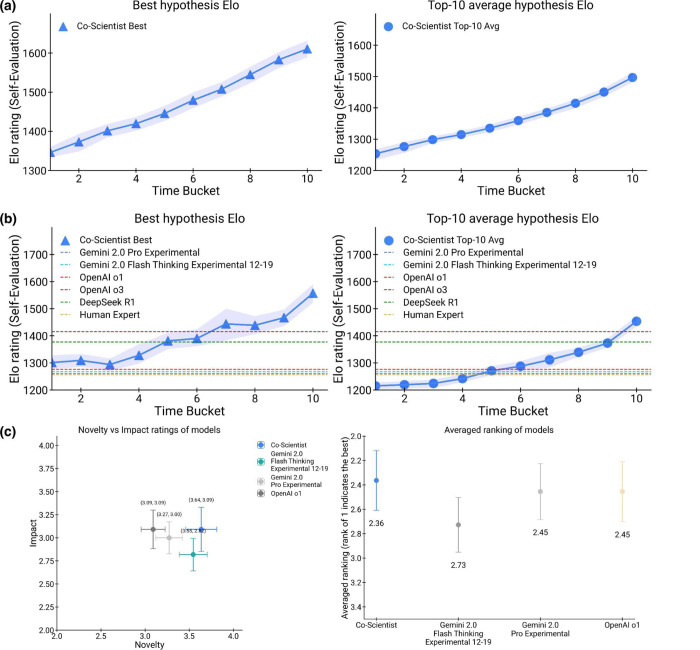
Scaling test-time compute enhances Co-Scientist’s scientific thinking and hypotheses quality. (a) Impact of test-time compute scaling on Co-Scientist hypotheses quality measured by Elo auto-evaluation: Generated hypotheses across 203 diverse scientific research goals were partitioned into ten sequential temporal buckets. The continuous upward trend in both the maximum individual Elo rating (left) and the top-10 average Elo rating (right) suggests the system’s capacity for self-improvement towards high quality scientific hypotheses for complex problems. (b) Auto-evaluation benchmarking against standard language models and human experts: Evaluated on a subset of 15 highly complex, expert-curated biomedical goals, Co-Scientist leverages test-time compute scaling to surpass and generate higher quality and novel hypotheses compared to human domain experts and state-of-the-art large language and reasoning models (e.g., OpenAI o1, o3-mini-high, DeepSeek-R1). (c) Blinded human expert evaluation: Independent domain experts rigorously assessed AI system hypotheses across 11 curated open biomedical problems. Left: Average expert ratings for hypothesis novelty and impact, evaluated independently on a 5-point Likert score and presented as grouped bar charts. Right: Overall expert preference ranking. Co-Scientist consistently achieved the highest expert ratings in novelty and impact and were selected as the preferred AI system by experts. For (a) and (b), data are presented as mean, and shaded regions indicate 95% confidence intervals. For two subfigures in (c), error bars represent probable errors, and the data are presented as mean ± probable errors. The exact sample sizes (n) are 203 research goals for (a), 15 goals for (b), and 11 goals for (c).

Co-Scientist’s research hypotheses and proposals were partitioned into ten temporal buckets of equal size. Each bucket corresponded to a sequential 10% of the total generation time with the first bucket containing the earliest 10% of generated Co-Scientist results, while the tenth bucket comprised the most recent 10%. For each bucket, we determined the average Elo rating of the top 10 hypotheses and the maximum individual Elo rating (the “best Elo”). These average and best Elo ratings were averaged across 203 research goals and their corresponding tournaments. The resulting performance trends as seen in Fig. 2a, across both metrics, serve as a measure of Co-Scientist’s quality improvement as it spends more time in thinking and computation—the most recent hypotheses demonstrate a significant quality enhancement compared to the initial ones. Notably, although the Elo rating is not the direct optimization target, its progressive increase emerges from the system’s information feedback loops that enable recursive self-improvement.

To further contextualize this observation, we focused our analysis on a subset of 15 research goals, curated as challenging problems by seven biomedical experts in their respective fields of expertise (Fig. 2b). These experts held doctoral degrees in biological or life science disciplines and were actively working as postdoctoral researchers or faculty members at academic research institutions. These research goals were consistently structured and formatted, encompassing a research title, a clear set of goals, preferences specifying biological or disease areas of interest, desirable solution attributes, and constraints on experimental techniques.

In addition to the research goals, the experts provided their “best guess” hypotheses or solutions. We then included outputs from other state-of-the-art LLMs and reasoning models at the time of this study (Gemini 2.0 Pro Experimental, Gemini 2.0 Flash Thinking Experimental 12–19, OpenAI o1, OpenAI o3-mini-high, and DeepSeek R1) in a tournament along with the expert “best guess” and Co-Scientist for each curated goal. Performance was assessed using Co-Scientist Elo rating metric.

The trends previously observed with test-time compute scaling in Fig. 2a were consistent within this subset. Furthermore, as shown in Fig. 2b, Co-Scientist eventually significantly surpassed the other frontier LLMs and reasoning models in Elo rating with iterative improvement. Notably, newer reasoning models, such as OpenAI o3-mini-high and DeepSeek R1, demonstrated competitive performance while requiring significantly less compute and reasoning time. Finally, we observed no evidence of performance saturation as measured by Elo, suggesting that further scaling of test-time compute in this paradigm could yield continued improvements in result quality of Co-Scientist as long as the research goal is tractable and benefits for the “search and explore paradigm”. It’s worth noting again that Co-Scientist architecture is model agnostic and is likely to benefit from further advancements in frontier and reasoning LLMs such as the most recent Gemini 3 models.

**Extended Data Fig. 1 |:**
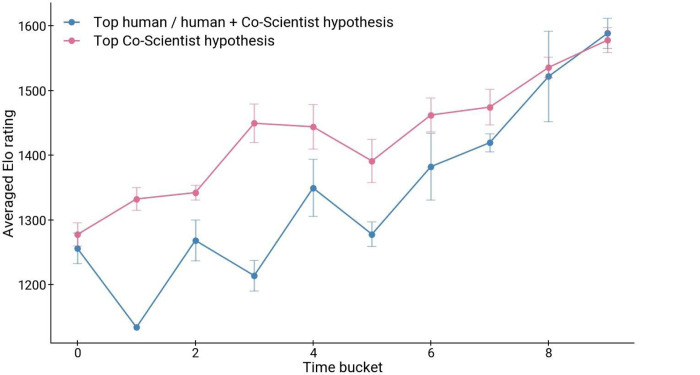
AI-augmented expertise with Co-Scientist through Elo-based auto-evaluation. Through its self-improvement process, Co-Scientist refines and enhances expert “best guess” solutions over time, as measured by the Elo rating on a subset of 15 curated research goals. It is important to note that the Elo metric is auto-evaluated and not based on independent ground truth. The error bar indicates the corresponding standard error of the mean (SEM). Data are presented as mean ± SEM for 15 independent expert-curated research goals.

Building upon Co-Scientist’s ability to refine and improve research hypotheses and proposals iteratively, we investigated its potential to improve upon expert “best guess” solutions. Consistent with our previous observations, Co-Scientist demonstrated the capacity to enhance expert’s “best guess” solutions over time, as evidenced by the Elo metric in Extended Data Fig. 1. Notably, the improvement trends initially mirrored those of Co-Scientist’s self-generated solutions but subsequently surpassed them as measured by Elo. While this is a preliminary finding requiring further validation, it suggests a new paradigm of human-AI collaboration in scientific discovery with systems such as Co-Scientist, augmenting and accelerating the work of expert scientists.

#### Co-Scientist yields potentially novel and impactful results for experts.

To obtain expert feedback and assess preferences, we conducted a small-scale expert evaluation on 11 of the 15 previously curated research goals. We asked the experts who curated the research goals to assess outputs from Co-Scientist, Gemini 2.0 Flash Thinking Experimental 12–19, Gemini 2.0 Pro Experimental, and OpenAI o1 models. Specifically, they provided a preference ranking (1 being most preferred and 4 being least preferred) and rated the novelty and impact of the proposed solutions on a 5-point scale, ranging from 1 (worst) to 5 (best) following this rubric:

- Novelty: Higher-ranked outputs should propose hypotheses that, to the best of the expert’s knowledge, have not been previously published in any form. Hypotheses similar to existing proposals, even with minor modifications, should rank lower, and exact replicas of previously proposed and performed experiments should receive the lowest ranking.
- Impact: Higher-ranked outputs should address significant open questions in the field and have the potential to substantially advance scientific understanding or lead to practical applications.

Across 11 expert-evaluated research goals, outputs generated by Co-Scientist were most preferred and rated higher in novelty and impact axes compared to the other baseline models. Specifically, Co-Scientist received an average preference rank of 2.36, and novelty and impact ratings of 3.64 and 3.09 (out of 5) as shown in Fig. 2c. These evaluations reflect subjective expert assessments, not objective ground truth. Notably, the human expert preferences also appear to be concordant with relative Elo ratings as can be inferred from Fig. 2b, 2c.

**Extended Data Fig. 2 |:**
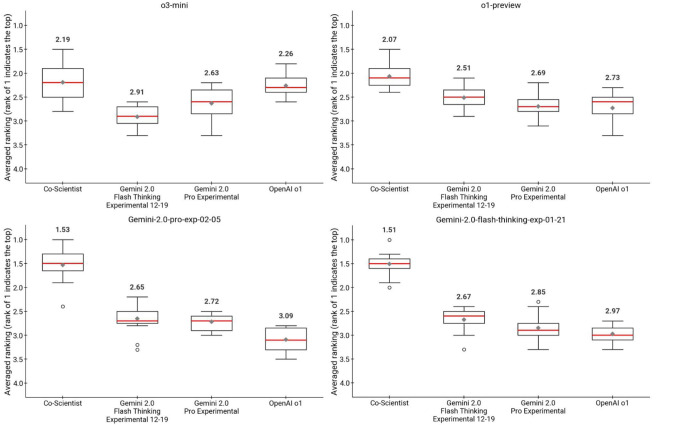
LLM preference ranking auto-evaluation of Co-Scientist and other baselines. Averaged preference ranking of results across 15 expert curated research goals generated by Co-Scientist, Gemini 2.0 Flash Thinking Experimental 12–19, Gemini 2.0 Pro Experimental, and OpenAI o1, using four different LLM evaluators: OpenAI o3-mini-2025–01-31 (upper left), OpenAI o1-preview-2024–09-12 (upper right), Gemini 2.0 Pro Experimental (lower left), and Gemini 2.0 Flash Thinking Experimental 01–21 (lower right). Lower numbers indicate better rankings. In each box plot, the central line represents the median ranking, the top and bottom edges of the box indicate the 25th and 75th percentiles respectively, the whiskers extend to the most extreme data points within 1.5 times the interquartile range, and the gray diamond marks the mean. Each box plot represents averaged evaluation scores for the 15 independent expert-curated research goals.

We also conducted the preference ranking evaluation for the 15 goals between Co-Scientist and other LLM and reasoning model baselines using the OpenAI o3-mini-2025–01-31, o1-preview-2024–09-12, Gemini 2.0 Pro Experimental and Gemini 2.0 Flash Thinking Experimental 01–21 as judges (LLM-as-a-judge evaluation). Co-Scientist outputs were the most preferred by all four evaluation judge LLMs as shown in Extended Data Fig. 2. Due to the small scale of these evaluations, further studies are necessary for any reliable conclusions. We further present a more comprehensive clinical expert evaluation focused on Co-Scientist proposals for drug repurposing in Supplementary Note 4 and and Supplementary Fig. 7.

### Real-world validations

#### Drug repurposing with Co-Scientist.

Rigorous assessment of a system’s ability to generate novel hypotheses for complex scientific problems necessitates end-to-end experimental validation. However, due to the challenging, time-consuming, and resource-intensive nature of such endeavors, large-scale experimental validation is infeasible. Instead, we selected areas of unmet clinical need to serve as a strong benchmark for the end-to-end system evaluation of the system’s hypotheses generation capability. Importantly, all experimental validations were conducted in collaboration with expert scientists, who provided guidance to Co-Scientist and prioritized wet-lab experiments.

Our first end-to-end validation of Co-Scientist is in drug repurposing, where the goal was to identify novel therapeutic indications for existing, approved drugs beyond their original use. This approach can accelerate the discovery of treatments for complex and rare diseases, as repurposed drugs have established safety profiles and are readily available. From a technical standpoint, this is a complex search and explore problem involving a large but finite set of drug-disease pairs as noted in Table 1.

Given Co-Scientist’s ability to synthesize and integrate information across a vast body of scientific and clinical literature, we hypothesized that drug repurposing would be an ideal test of the system’s capabilities. The validation of Co-Scientist’s predictions was performed using a multi-faceted approach, incorporating computational biology analyses, oncologist expert feedback, and *in vitro* wet-lab experiments using cancer cell lines.

We constrained Co-Scientist to explore potential repurposing hypotheses from a curated list of 2300 approved drugs across 34 cancer types and conducted an oncologists’ expert review of the predictions (Supplementary Note 5.1 and Supplementary Fig. 8). Building upon the positive feedback from clinical experts, we conducted *in vitro* wet-lab validation experiments for drug repurposing hypotheses generated by Co-Scientist for acute myeloid leukemia (AML), an aggressive hematological malignancy marked by uncontrolled proliferation of myeloblasts, resulting in impaired hematopoiesis. Disease recurrence remains associated with a critical unmet need for effective therapeutic options ^22^. The cell line based experiments conducted here serve as an initial biological validation step for Co-Scientist hypotheses, with intentionally straightforward methodology following established protocols. We selected four AML cell lines for covering different AML subtypes (MOLM-13, KG-1a, HL-60, NOMO-1), and a non-AML cell line (TK6), based on the rationale provided by Co-Scientist and the clinical expert in the loop (Supplementary Note 5.2). It is important to emphasize that these wet-lab experiments function as a viability check of the drug repurposing pipeline, yet they are not a replacement for the rigorous pre-clinical and clinical assessment typically required for therapeutic validation. They provide an efficient biological reality check allowing us to rapidly evaluate AI-generated hypotheses before committing to more resource-intensive validation studies necessary for clinical translation.

#### Wet-lab validation of Co-Scientist AML drug repurposing candidates.

The candidate selection for wet-lab experiments was performed with meticulous expert oversight. Thirty top-ranked drug candidate hypotheses were shared with expert oncologists (an example detailed Co-Scientist output is provided in Supplementary Note 6). The experts evaluated the hypotheses, selecting drug candidates based on their potential to modulate key molecular signaling pathways associated with disease progression and resistance.

The primary selection criteria prioritized compounds with multi-pathway activity, specifically those targeting dysregulated inflammatory signaling, metabolic reprogramming, and aberrant cell proliferation. Emerging evidence indicates that these interconnected biological processes play critical roles in AML relapse and treatment resistance ^23^. Candidates were further prioritized based on preclinical mechanistic insights demonstrating relevance to AML biology, including their predicted effects on leukemic cell survival, interactions within the bone marrow microenvironment, and mechanisms underlying therapeutic resistance.

Based on potential mechanisms of action, five initial drug repurposing candidates, Binimetinib, Pacritinib, Cerivastatin, Pravastatin, and Dimethyl fumarate (DMF), were selected for further wet-lab validation in AML. Drug details are listed in Supplementary Note 5.3.

**Extended Data Fig. 3 |:**
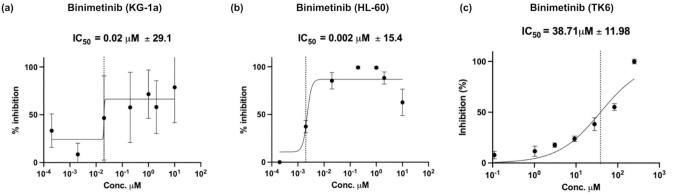
Dose response curves of drug repurposing candidate Binimetinib in other cell lines. Binimetinib demonstrates activity inhibiting cell viability in KG-1a, HL-60, and TK6 cell lines. X-axis is the drug concentration (μM), and Y-axis is the percentage of growth inhibition. Binimetinib’s target is related to the RAS–RAF–MEK–ERK pathway which is not generally expected to be overactive or essential in the TK6 cell line compared to AML cells. Thus, the significantly higher IC_50_ in TK6 compared to three AML cell lines (MOLM-13, KG-1a, HL-60), is consistent with its intended mechanism of action. Data are presented as mean ± SD of n = 3 biologically independent experiments.

**Fig. 3 |:**
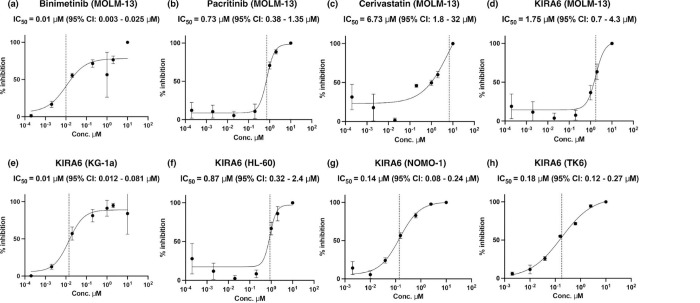
*In vitro* biological validation of Co-Scientist generated single-agent repurposing candidates for acute myeloid leukemia (AML). Co-Scientist identified promising biologically active compounds, ranging from candidates with existing preclinical rationales to completely novel therapeutic targets for AML. (a-c) Dose-response curve of MOLM-13 AML cells treated with Binimetinib, Pacritinib, and Cerivastatin. Co-Scientist nominated candidates with existing evidence, demonstrating potent anti-leukemic activity. (d-h) Dose-response curves of the completely novel Co-Scientist predicted candidate KIRA6 (an IRE1α inhibitor) evaluated in different AML cell lines (d-g) and the normal lymphoblastoid control cell line TK6 (h). KIRA6 exhibits highly selective cytotoxicity against the KG-1a AML cell line compared to the non-malignant TK6 control. The 18-fold separation establishes a promising *in vitro* therapeutic window and suggests Co-Scientist’s promising capability to search, reason and identify biologically active compounds. The X-axis represents drug concentration (μM) on a logarithmic scale, and the Y-axis represents the percentage of growth inhibition. Data are presented as mean ± SD of n=3 biologically independent experiments. Exact IC_50_ values were determined using non-linear regression curve fitting.

Of the five drugs tested (experimental setup detailed in Supplementary Note 5.4), Binimetinib, Pacritinib, and Cerivastatin demonstrated inhibition of cell viability (Fig. 3a–c). Notably, Binimetinib, which is already approved for the treatment of metastatic melanoma, exhibited an half-maximal inhibitory concentration (IC_50_) as low as 2 nM in all AML cell lines (except NOMO-1), but much higher in the TK6 non-AML cell line (Fig. 3a and Extended Data Fig. 3). While Binimetinib demonstrated limited efficacy as monotherapy in heavily pretreated, RAS-mutant relapsed/refractory AML in a prior phase II study, Co-Scientist proposed an alternative repurposing strategy for frontline treatment without molecular profiling, based on modulation of baseline RAS-MEK-ERK pathway activity that may influence sensitivity to conventional chemotherapy in treatment-naive disease ^24^. This result shows the promise of Co-Scientist to aid with drug repurposing.

#### Novel single-agent AML repurposing candidates.

We next investigated Co-Scientist’s capacity to autonomously propose novel single-agent drug repurposing candidates without oversight. Towards this, the system was directed to generate a ranked list of repurposing candidates for AML that were not previously repurposed for the target indication and without any prior preclinical evidence. Further, the Co-Scientist did not receive any additional external inputs, such as the DepMap scores or human expert feedback. We then determined if these novel candidates suggested by Co-Scientist could be validated in the laboratory.

For *in vitro* laboratory validation, the domain experts reviewed and selected the top three repurposing candidates, to treat AML: Nanvuranlat, KIRA6, and Leflunomide.

The detailed Co-Scientist output, including the hypothesis, rationale and self-generated novelty review, is provided for KIRA6 in Supplementary Note 6. As can be seen, the system identifies that targeting IRE1α in the context of AML has been explored ^25^ before but not with the specific drug, KIRA6 suggesting the system is reasonably well-calibrated in its assessment of novelty. The system suggests an overall moderate level of novelty for the hypothesis.

**Extended Data Fig. 4 |:**
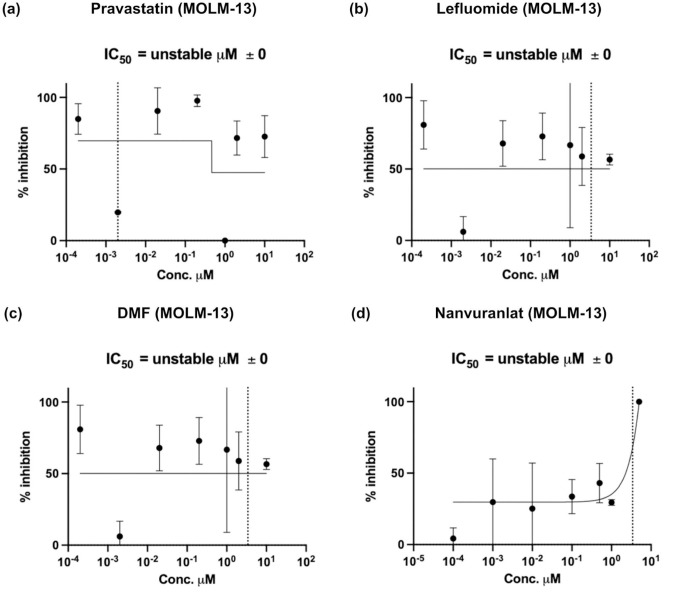
Dose response curves of the drug repurposing candidates with little to no effect on MOLM-13. Of the expert-selected drug repurposing candidates, Pravastatin and DMF showed little to no effect on the MOLM-13 cell line across the concentrations tested. Of the novel drug repurposing candidates, Leflunomide and Nanvuranlat showed little to no effect on the MOLM-13 cell line across the concentrations tested. X-axis is the drug concentration (μM), and Y-axis is the percentage of growth inhibition. Data are presented as mean ± SD of n = 3 biologically independent experiments.

Of the three drugs tested, treatment with the IRE1α inhibitor KIRA6 showed inhibition of cell viability in several AML cell lines representing different molecular subtypes, KG-1a, MOLM-13, HL-60, NOMO-1, and a non-AML cell line, TK6, as a control (Fig. 3d–h, Supplementary Note 5.2, Supplementary Table 4). IC_50_ values of KIRA6 were all in nM or low μM range, but significantly more effective in KG-1a cells, which had an IC_50_ of 10 nM, compared to the non-AML control cell line, TK6, which had an IC_50_ of 180 nM. KIRA6 was also slightly more effective in NOMO-1 cells, with an IC_50_ of 144 nM, but notably, MOLM-13 and HL-60 cells were markedly less sensitive to KIRA6, with IC_50_ values of 1750 nM and 870 nM, respectively. The 18-fold separation between the highly primitive KG-1a cells and the normal lymphoblastoid TK6 line highlights a potential selective therapeutic window. The differential sensitivities observed across distinct AML subtypes indicate that IRE1α blockade may be most effective in targeting primitive, stem-like AML populations over more differentiated lineages. Comprehensive cytogenetic rationales and molecular mechanisms correlating these varying cell line sensitivities to the IRE1α–XBP1 axis are detailed in Supplementary Note 5.2. Nanvuranlat and Leflunomide instead show limited effect on MOLM-13 cells (Extended Data Fig. 4).

#### Novel synergistic AML drug combinations.

**Extended Data Fig. 5 |:**
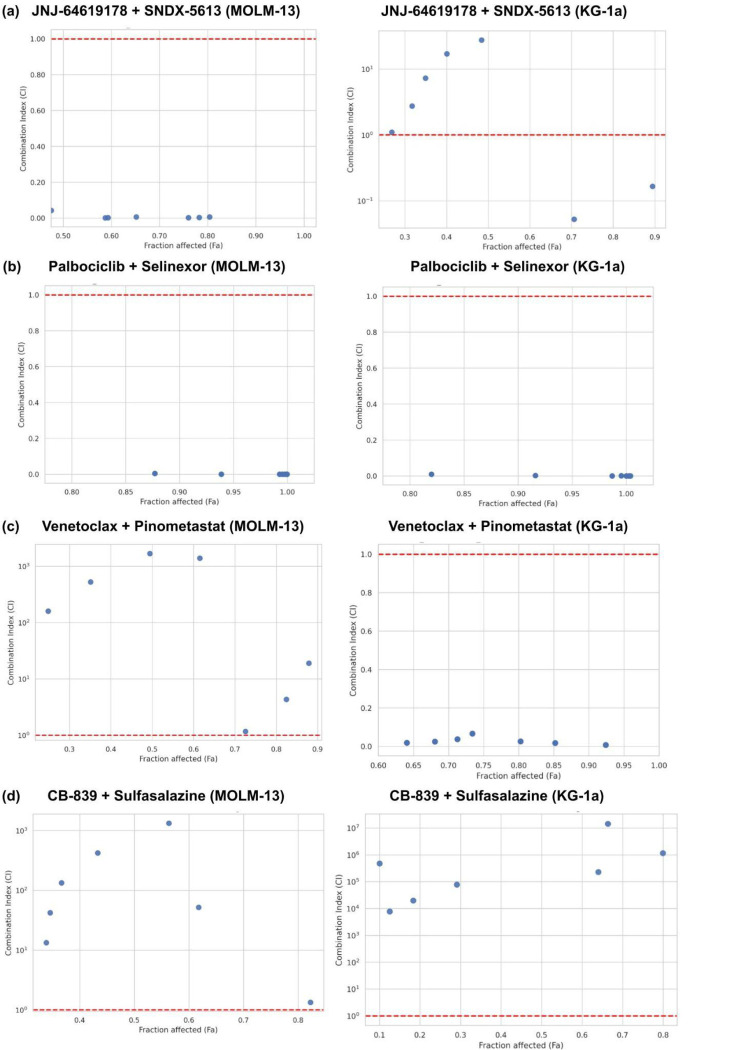
Comprehensive synergy analysis of Co-Scientist nominated dual drug combinations in AML cell lines. Building upon the representative example in Fig. 4a–b, this figure details the pharmacological interactions of additional AI-predicted doublets evaluated in MOLM-13 and KG-1a cell lines. Synergy was quantified using the Chou-Talalay method based on fixed-ratio dose-response data. Each panel presents a Fraction affected versus Combination Index (Fa-CI) plot. The horizontal red dashed line indicates a strictly additive effect (CI = 1.0). Data points below the line (CI < 1.0) indicate synergistic interactions, while points above the line (CI > 1.0) indicate antagonism. The results highlight that while certain combinations (e.g., Palbociclib + Selinexor) exhibit consistent synergy across different genetic backgrounds, others display context-dependent interaction profiles strongly influenced by the specific cell line’s mutational status.

**Extended Data Fig. 6 |:**
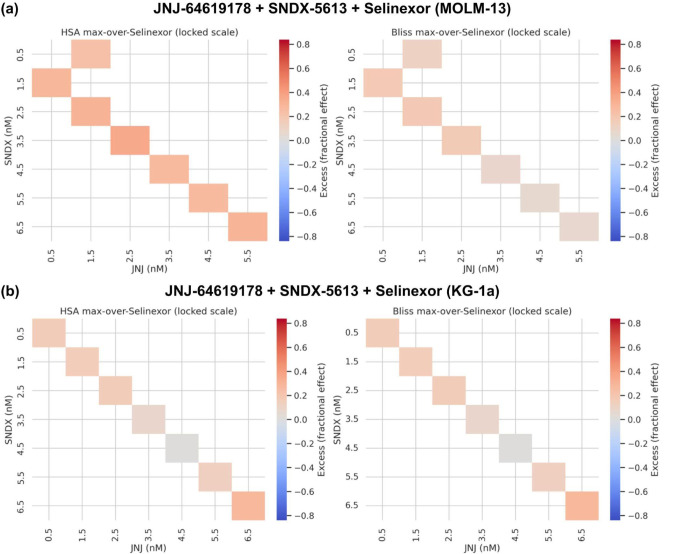
Excess fractional effect heatmaps for Co-Scientist predicted triple drug combinations. Complementing Fig. 4c–d, this figure provides the interaction matrices for the other evaluated triplet regimen (JNJ-64619178 + SNDX-5613 + Selinexor) across AML cell lines. Synergy and antagonism are quantified across a comprehensive grid of drug concentrations (nM) utilizing both the Highest Single Agent (HSA) and Bliss independence models. The heatmaps display the fractional excess effect: the difference between the observed combined empirical effect and the predicted additive effect. Positive excess effects are visualized in red (synergy), zero excess in white (additivity), and negative excess in blue (antagonism). To compress the three-dimensional dose space into a two-dimensional matrix, the plots display the maximum positive interaction across the third drug’s dose range.

**Fig. 4 |:**
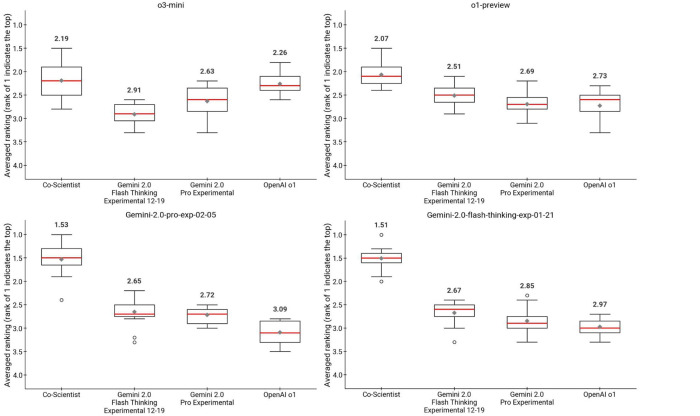
Validation of Co-Scientist predicted synergistic multi-drug combinations for acute myeloid leukemia (AML). Co-Scientist successfully navigated high-dimensional combinatorial spaces to propose effective multi-drug therapy regimens, validated here in the MOLM-13 and KG-1a AML cell lines. (a, b) Quantitative synergy analysis of the dual combination JNJ-64619178 and Selinexor. The plot illustrates the relationship between the Combination Index (CI) and the fraction affected (Fa) using the Chou-Talalay method. The horizontal red dashed line represents strictly additive effects (CI = 1.0). Data points falling below this threshold denote the synergy zone (CI < 1), while points above denote the antagonism zone (CI > 1), confirming strong synergistic interactions for this Co-Scientist proposed double drug combination. (c, d) Excess fractional effect heatmap for the triple drug combination JQ1, Olaparib, and MSA2. Synergy is quantified across a matrix of drug concentrations (nM) using the Highest Single Agent (HSA) and Bliss independence models. The color scale illustrates the deviation from predicted additive effects: red regions indicate a positive excess effect (synergy), while blue regions represent a negative excess effect (antagonism). These results suggest Co-Scientist’s ability to identify highly active, complex combinatorial treatments without exhaustive empirical screening. Complete interaction profiles are available in Extended Data Figs. 5, 6 and Extended Data Tables 1, 2. For all synergy analyses, experiments were performed in n = 3 biologically independent replicates.

A common strategy for more effective treatment is the combination of drugs that synergistically target different disease pathways, but searching and screening for these combinations becomes exponentially more complex as the number of drugs increases. This is a complex task even for experts; at the same time, it may be well suited for AI. To investigate this, we tasked Co-Scientist to identify promising synergistic multi-drug regimens for AML. We then evaluated seven drug combinations proposed by Co-Scientist in MOLM-13 and KG-1a cell lines. In MOLM-13 cells, responses were predominantly synergistic across both dual (e.g., JNJ-64619178 + Selinexor) and triple combinations (e.g., JQ1 + Olaparib + MSA2). In contrast, KG-1a cells exhibited highly context-dependent responses with a mixture of synergy and antagonism, likely reflecting their distinct chemoresistant molecular profile (TP53-mutant). A comprehensive summary of all interaction patterns is provided in Fig. 4, Extended Data Figs. 5 and 6, Extended Data Tables 1, 2, and Supplementary Note 5.5. These patterns likely reflect the underlying molecular profiles of the two cell lines (detailed in Supplementary Note 5.3 and 5.5). Further mechanistic studies will be required to define the molecular determinants of response to combination therapy across AML subtypes, and to identify predictive biomarkers that could enable rational regimen selection.

These results not only demonstrate Co-Scientist’s ability to identify potent single agents but also highlight its utility in proposing novel, synergistic drug combinations that have potential in addressing therapeutic resistance and treatment-refractory disease. Importantly, it shows the potential of Co-Scientist to discover novel drug combinations without the requirement of large-scale wet-lab screening, which becomes exponentially more costly and difficult as larger combinations are considered. Therefore, Co-Scientist may enable and unlock capabilities that were previously restricted by wet-lab design and feasibility.

#### Guiding clinical translation design.

While the results are promising, translating these predictions from Co-Scientist into clinical practice will be highly challenging, as the complexity of a disease model, patient heterogeneity, and disease variability cannot be fully captured in such limited *in vitro* experiments. Even if a hypothesis generated by Co-Scientist is well-reviewed by oncologists and supported by preclinical rationale and strong *in vitro* experiments, this does not guarantee *in vivo* efficacy or clinical success. Factors such as drug bioavailability, pharmacokinetics, off-target effects, and patient selection criteria can all impact onward clinical trial outcomes. Moreover, in case of hematological malignancies, the tumor microenvironment and systemic interactions may introduce unforeseen resistance mechanisms, further complicating translation from hypothesis to clinical benefit.

To more faithfully approximate the parameters that govern real-world therapeutic decision-making, we tasked Co-Scientist with a structured translational analysis using a detailed clinical variable framework encompassing patient demographics, ELN2022 risk stratification, molecular features, preclinical activity, and established safety and PK/PD data (Supplementary Note 5.6).

Here for example, Co-Scientist’s structured translational analysis successfully identified a specific clinical niche for Binimetinib: frail, heavily pretreated AML patients. The system accurately deduced that Binimetinib’s unique metabolic pathway (UGT1A1) circumvents severe CYP3A4-dependent drug-drug interactions common with azole antifungals—a major limitation for current targeted therapies (detailed in Supplementary Note 5.6). Extended results and methodological details are provided in Supplementary Note 5.2 and 5.6. Taken together, these analyses illustrate how Co-Scientist can help clinician scientists move beyond initial hypothesis generation to synthesize diverse clinical and biological variables into testable, clinically grounded therapeutic strategies.

#### Uncovering novel therapeutic targets for liver fibrosis.

Co-Scientist utilized a method employing human hepatic organoids coupled with live cell imaging to find novel therapeutic targets for severe liver fibrosis ^1, 26, 27^. Co-Scientist was tasked with generating hypotheses on target epigenetic alterations (three top-ranked were selected by experts) and identifying drugs targeting these predicted epigenetic modifiers. It successfully identified three novel epigenetic modifiers and drugs targeting them, and two of them exhibited significant anti-fibrotic activity in the hepatic organoids without causing cellular toxicity. Critically, one of the effective drugs (Vorinostat) is already FDA-approved for another cancer indication, creating an opportunity for drug re-purposing for liver fibrosis treatment ^1^. This example also highlights the potential of AI systems such as Co-Scientist to make unexpected connections across disparate disciplines and diseases (cancer and liver fibrosis) and synthesize novel, helpful and impactful hypotheses and discoveries.

#### Recapitulating a breakthrough in antimicrobial resistance.

Co-Scientist was also challenged to independently discover the mechanism behind the broad host range and rapid spread of capsid-forming phage-inducible chromosomal islands (cf-PICIs), mobile elements that carry virulence and antibiotic resistance genes across diverse bacterial species (including *E. coli* and *K. pneumoniae*). With only minimal background information, Co-Scientist independently and accurately proposed the groundbreaking, top-ranked hypothesis that cf-PICIs interact with diverse phage tails to expand their host range ^2^. This finding, generated by the AI in just two days, precisely matched the primary discovery of an independent, co-timed genomic and experimental study prior to completing peer-review ^21^. This convergence and recapitulation is another demonstration of Co-Scientisťs potential to accelerate scientific discovery by synthesizing complex scientific information and generating rigorous scientific hypotheses on-par with experts.

## Discussion

In this work, we report the development and initial validation of a multi-agent, Gemini based AI system, Co-Scientist, designed as a structured scientific thinking engine to accelerate novel scientific discovery. Co-Scientist moves beyond conventional computational approaches through the in-silico implementation of a multi-agent architecture that mirrors the core aspects of the scientific method. Instead of brute-force generation, the system iteratively refines hypotheses through a “generate, debate, evolve” paradigm. This method, which incorporates self-debate, tournament-based selection, and iterative evolution and refinement, enables a progressive convergence on high-quality, well-supported hypotheses, thereby scaling research ideation with test-time compute rather than exhaustive generation. The system’s context memory, combined with the iterative self-improvement cycle, functions as an emergent internal model of the scientific research process. While not an explicit symbolic model, it represents a progressively more coherent and interconnected state of knowledge, facilitating the synthesis of information and the identification of knowledge gaps.

The practical utility of this approach was demonstrated through the generation of novel and experimentally tractable hypotheses across three challenging and varied biomedical problems. In oncology, Co-Scientist identified drug repurposing candidates for AML that showed *in vitro* efficacy at clinically relevant concentrations. For liver fibrosis, it proposed novel epigenetic targets, leading to the experimental validation of several anti-fibrotic compounds, including one FDA-approved drug. Furthermore, in microbiology, the system independently recapitulated a novel, (and then) unpublished mechanism of mobile genetic element transfer between bacteria. These findings provide preliminary evidence that Co-Scientist can contribute meaningfully to scientific discovery by amplifying scientists.

This system’s architecture is model-agnostic, allowing it to leverage the advancing capabilities of frontier LLMs without requiring retraining of the whole agentic framework such as Gemini 3, GPT 5.4 and Opus 4.6. With the latest advances in frontier models, we expect further significant improvements in the quality of hypotheses generated and the complexity of scientific tasks the system can autonomously accomplish.

Despite these promising early results, several limitations must be addressed. Co-Scientist’s knowledge is constrained by its reliance on open-access scientific literature, which may lead to the omission of critical prior art behind paywalls and a systemic lack of access to negative experimental results. Furthermore, the quality of generated hypotheses relies on the mixed and contradictory quality of the source literature; thus, there is a risk of propagating erroneous or irreproducible findings. A key future direction is the development of agents with enhanced provenance capabilities to trace claims to specific figures or data within a source, mitigating the impact of unreliable literature.

Co-Scientist also inherits the intrinsic limitations of its underlying models, including imperfect factuality and the potential for hallucinations. Improving reasoning capabilities is a critical area for future work. Additionally, the validation of Co-Scientist’s hypotheses, while successful, remains preliminary.

Finally, the broader integration of such AI systems into the scientific workflow requires careful consideration of potential bias, which could risk diminishing critical thinking or homogenizing research directions. While AI has the potential to democratize access to scientific information, particularly in resource-limited settings, it is essential to develop robust verification methods and maintain rigorous peer review to ensure that AI can augment, rather than replace, human scientific reasoning and creativity. Improper use of such AI systems without rigorous peer-review and guardrails could also lead to worsening of the scientific reproducibility crisis through production of low quality scientific artifacts. Further details regarding safety and ethical implications are provided in Supplementary Note 7.

The continued development of Co-Scientist will focus on three key areas. Immediate improvements will target the system’s robustness by enhancing learning and knowledge base, literature search capabilities to broaden access, implementing more rigorous fact-checking against external databases and tools, and improving citation recall. Future advancements will focus on expanding the system’s core capabilities. This includes integrating agents that can directly reason over public databases and multimodal data, enabling bioinformatics and data science tasks. The implementation of reinforcement learning from human and experimental feedback could further optimize the hypothesis generation and refinement process.

Expanded evaluations are also necessary to assess Co-Scientist’s generalizability across a wider range of scientific disciplines. This requires developing more objective and automated evaluation metrics that move beyond current ranking systems and engaging a larger cohort of domain experts to stress-test the system with diverse and complex research queries.

In the fullness of time, integrating Co-Scientist with laboratory automation platforms could create a closed-loop, autonomous system for hypothesis generation, experimental validation, and iterative learning, significantly accelerating the pace of scientific discovery.

## Conclusion

Co-Scientist represents a promising step towards AI-assisted augmentation of scientists and acceleration of scientific discovery. Its ability to think scientifically, generate novel testable hypotheses across diverse scientific and biomedical domains, some supported by experimental findings, along with the capacity for recursive self-improvement with increasing compute, demonstrates the promise of meaningfully accelerating scientists’ endeavors to resolve grand challenges in human health, medicine and science. This innovation opens numerous questions and opportunities. Applying the empiric and responsible approach of science to Co-Scientist itself can thereby enable safe exploration of its undoubted potential, including how collaborative and human-centred AI systems might be able to augment human ingenuity and accelerate scientific discovery.

## Methods

### Overview of Co-Scientist architecture.

Co-Scientist employs a multi-agent architecture built upon Google’s Gemini. In this study, we used Gemini 2.0 models as the base foundational LLM for all agents ^14^, integrated within an asynchronous task execution framework. This framework allows for flexible scaling of test-time compute resources, facilitating advanced scientific thinking and reasoning. Given a research goal specified by an expert scientist in natural language, Co-Scientist generates hypotheses that adhere to the following default criteria: (1) **Alignment with the provided research goal.** The generated outputs must precisely align with the research goals, preferences and constraints defined by the scientist. (2) **Plausibility.** The system outputs should be free of readily apparent flaws. Any potential contradictions with prior literature or established knowledge must be explicitly stated and justified. (3) **Novelty.** A key objective of Co-Scientist is to generate novel hypotheses, conjectures, and research plans grounded in prior literature, rather than simply synthesizing existing information (a capability already addressed by existing “deep research” tools ^28^). (4) **Testability.** The system outputs should be amenable to empirical validation within the constraints specified by the scientist. (5) **Safety.** The system outputs will be controlled to prevent enabling unsafe, unethical, or harmful research. Aside from these default criteria, Co-Scientist can be configured with additional criteria, preferences, and constraints as needed. For instance, it can be configured to generate outputs in formats preferred by the researcher to improve interpretability and readability.

At a high level, Co-Scientist comprises four key components: (1) **Natural language input-output (IO) interface.** Scientists interact with and supervise the system primarily through natural language. This allows them to not only define the initial research goal but also refine it at any time, provide feedback on generated hypotheses (including their own solutions), and generally steer and guide the system’s progress. (2) **Asynchronous task framework.** Co-Scientist employs a multi-agent system where specialized agents operate as worker processes within an asynchronous, continuous, and configurable task execution framework. A dedicated Supervisor agent manages the worker task queue, assigns specialized agents to these processes, and allocates resources. This design enables the system to flexibly and effectively utilize computational resources and iteratively improve its scientific reasoning and quality of hypotheses. (3) **Specialized agents.** Following inductive biases and scientific priors derived from the scientific method, the process of scientific reasoning and hypothesis generation is broken down into sub-tasks. Individual, specialized agents, each equipped with customized instruction prompts, are designed to execute these sub-tasks. These agents operate as workers coordinated by the Supervisor agent. (4) **Context memory.** In order to enable iterative computation and scientific reasoning over long time horizons, Co-Scientist uses a persistent context memory to store and retrieve states of the agents and the system during the course of the computation. The specific Co-Scientist design was arrived at with iterative developments and feedback from expert scientists and is reflective of the current capabilities of the underlying LLMs. Co-Scientist multi-agent architecture is depicted and summarized in Fig. 1b.

Throughout the following section, we employ a recurring example: generating hypotheses for exploring the biological mechanisms of Amyotrophic Lateral Sclerosis (ALS) to illustrate the various components of Co-Scientist. While this example has been reviewed by domain experts, it remains illustrative and may contain errors. Importantly, this example does not aim to suggest potential therapeutic avenues for ALS and should be interpreted with utmost caution. We have also provided the pseudocode demonstrating agent logic in Supplementary Note 8. All the prompts used in the agents are listed in Supplementary Note 9, and all the examples are listed in the Supplementary Note 10.

### From research goal to research plan configuration.

The research goal, specified by the scientist, serves as the entry point to Co-Scientist. Leveraging the multimodal and long context capabilities of Gemini models, Co-Scientist efficiently processes research goals of varying complexity, from simple statements to extensive documents spanning tens of thousands of natural language tokens or other relevant data (e.g., including hundreds of prior publication PDFs). The research goal may also incorporate specific constraints, attributes, and preferences related to the scientist’s particular laboratory setting or field of work.

Co-Scientist then parses the goal to derive a research plan configuration for generating research proposals. This configuration captures the desired proposal preferences, attributes, and constraints. For example, it specifies whether Co-Scientist should exclusively propose novel hypotheses. It also specifies the criteria for evaluating hypothesis quality, such as novelty and experimental feasibility. These criteria are then used by the system during its auto-evaluation, tournament debates and self-improvement phases. The attributes, preferences, and evaluation criteria can all be customized to a given research goal. To illustrate this process, we present an example research goal and its corresponding parsed research plan configuration in Supplementary Note 10.1, where the goal is to develop a novel hypothesis related to phosphorylation of the Nuclear Pore Complex (NPC) as a causative mechanism for ALS ^29^.

Based on the research plan configuration, the Supervisor agent initiates the creation of a task queue and begins orchestrating the specialized agents. The system operates continuously and asynchronously. Periodically, the Supervisor agent calculates a comprehensive set of summary statistics, reflecting the system’s state and progress toward the specified research goal. These statistics inform decisions regarding resource allocation and the determination of whether a terminal state for the overall computation has been reached. The state is periodically written to the associated context memory of the system and leveraged as feedback in subsequent rounds of computation. It also enables easy restarts in case of any failure in the system components.

### The specialized agents underpinning Co-Scientist.

At the heart of Co-Scientist are a coalition of specialized agents, each orchestrated by the Supervisor agent. These agents are designed to emulate the scientific reasoning process, enabling them to generate novel hypotheses and research plans. Each agent is provided a “library of strategies”, (i.e. a collection of prompts) to better explain and help it perform its assigned task. They are also equipped to interact with and utilize external tools, such as web search engines and specialized AI models, through application programming interfaces (APIs). These specialized agents are enumerated below: (1) **Generation agent.** The agent initiates the research process by generating the initial focus areas, iteratively extending them and generating a set of initial hypotheses and proposals that address the research goal. This involves exploring relevant literature using web search, synthesizing existing findings into novel directions, and engaging in simulated scientific debates for iterative improvement. (2) **Reflection agent.** This agent simulates the role of a scientific peer reviewer, critically examining the correctness, quality, and novelty of the generated hypotheses and research proposals. Furthermore, it evaluates the potential of each hypothesis to provide an improved explanation for existing research observations (identified via literature search and review), particularly those that may be under-explained. (3) **Ranking agent.** An important abstraction in Co-Scientist is the notion of a tournament where different research proposals are evaluated, debated and ranked enabling iterative improvements. The Ranking agent employs and orchestrates an Elo-based tournament ^30^ to assess and prioritize the generated hypotheses at any given time. This involves pairwise comparisons, facilitated by simulated scientific debates, which allow for a nuanced evaluation of the relative merits of each hypothesis. (4) **Proximity agent.** This agent asynchronously computes a proximity graph for generated hypotheses, enabling clustering of similar ideas, de-duplication, and efficient exploration of the hypothesis landscape. (5) **Evolution agent.** Co-Scientist’s iterative improvement capability relies heavily on this agent, which continuously refines the top-ranked hypotheses emerging from the tournament. Its refinement strategies include synthesizing existing ideas, using analogies, leveraging literature for supporting details, exploring unconventional reasoning, and simplifying concepts for clarity. (6) **Meta-review agent.** This agent also enables Co-Scientist’s continuous improvement by synthesizing insights from all reviews, identifying recurring patterns in tournament debates, and using these findings to optimize other agents’ performance in subsequent iterations. This also enhances the quality and relevance of generated hypotheses and reviews in subsequent iterations. At the end of the Co-Scientist computation process, this agent also synthesizes top-ranked hypotheses and reviews into a comprehensive research overview for review by the scientist.

The Supervisor agent’s seamless orchestration of these specialized agents enables the development of valid, novel, and testable hypotheses and research plans tailored to the input research goal provided by the scientist.

In summary, the Generation agent curates an initial list of research hypotheses satisfying a research goal. These are then reviewed by the Reflection agent and evaluated in a tournament by the Ranking agent orchestrating debate matches involving the hypothesis. The Evolution, Proximity, and Meta-review agents operate on the tournament state to help improve the quality of the system outputs.

The Supervisor agent periodically computes and writes to the context memory, a comprehensive suite of statistics, including the number of hypotheses generated and requiring review, and the progress of the tournament. These statistics also include analyses of the effectiveness of different hypothesis generation methodologies (e.g., generating new ideas via the Generation agent vs. improving existing ideas via the Evolution agent). Based on these statistics, the Supervisor agent then orchestrates subsequent system operations, i.e., generating new hypotheses, reviews, tournaments, and improvements to existing hypotheses, by strategically weighting and sampling the specialized agents for execution via the worker processes.

Importantly, the Meta-review agent enables feedback propagation and learning without back-propagation techniques (e.g., fine-tuning or reinforcement learning) ^31^. The Meta-review agent generates feedback applicable to all agents, which is simply appended to their prompts in the next iteration—a capability facilitated by the long-context search and reasoning capabilities of the underlying Gemini models. Through this feedback loop, Co-Scientist continuously learns and improves in subsequent iterations with more compute scaling.

Finally, while our work leverages Gemini 2.0, Co-Scientist framework is model-agnostic and portable to other similar models or combinations thereof. Future LLM improvements like our Gemini 3 models will further enhance Co-Scientist’s capabilities to generate novel scientific hypotheses and perform complex tasks over long time horizons.

#### Generation agent.

The Generation agent employs a diverse array of strategies (encoded as prompts), techniques and tools to generate novel hypotheses, such as the following:

- **Literature exploration via web search.** The agent iteratively searches the web, retrieves and reads relevant research articles, learns about topics and grounds its reasoning by summarizing prior work and building a knowledge base of scientific facts. It then builds on this summary to generate novel hypotheses and research plans. An example prompt is given in Supplementary Note 9.1.
- **Simulated scientific debates.** Here, the Generation agent simulates scientific debates among experts by employing self-critique and self-play techniques. These debates typically involve multiple turns of conversations leading to a refined hypothesis generated at the end. An example prompt is given in Supplementary Note 9.1.
- **Iterative assumptions identification.** The agent iteratively identifies testable intermediate assumptions, which, if proven true, can lead to novel scientific discovery. These plausible assumptions and their sub-assumptions are identified through conditional reasoning hops and subsequently aggregated into complete hypotheses.
- **Research expansion.** To identify previously unexplored areas of the hypothesis space, the Generation agent reviews existing hypotheses and the research overview and feedback provided by the Meta-review agent in the previous iteration. This is used to inform additional exploration directions in the research hypothesis space.

An example hypothesis and research proposal output from the Generation agent is presented in Supplementary Note 10.2 for the aforementioned research goal regarding explaining a basic mechanism related to ALS. The Generation agent also summarizes and categorizes each generated hypothesis, allowing scientists to quickly grasp the core ideas.

#### Reflection agent.

Reviews are integral to Co-Scientist’s effectiveness in generating novel proposals. The Reflection agent searches relevant prior work and data (via web search or a dedicated scientist-provided repository), assesses existing experimental evidence for or against a given hypothesis, and rigorously verifies the novelty, correctness, and quality of generated outputs with tools. Effective reviews filter inaccurate and, when stipulated, non-novel hypotheses. Moreover, they also provide feedback to all other agents, driving continuous improvement. The Reflection agent employs the following types of review strategies:

- **Initial review.** Building on Co-Scientist’s default evaluation criteria, the Reflection agent performs an initial review assessing the correctness, quality, novelty, and a preliminary assessment of safety (ethics) of the generated hypotheses. For a more in-depth discussion on safety considerations see Supplementary Note 7. This initial review, which doesn’t use external tools like web search, aims to quickly discard flawed, non-novel, or otherwise unsuitable hypotheses.
- **Full review.** If a hypothesis passes the initial review, the Reflection agent performs a full review, leveraging external tools and web searches to identify relevant articles for improved reasoning and grounding. This review evaluates the hypothesis's correctness, quality, and novelty similar to the initial review but with full literature search. For correctness and quality, the agent scrutinizes underlying assumptions and reasoning. For novelty, it summarizes known aspects of the hypothesis and then judges their novelty based on existing literature. An example full novelty review, review critiques, and a complete full review example are shown in Supplementary Note 10.3, 10.4 and 10.5, respectively.
- **Deep verification review.** The Reflection agent also conducts a deep verification review, decomposing the hypothesis into constituent assumptions. Each assumption is further broken down into fundamental sub-assumptions, decontextualized, and independently evaluated for correctness to identify invalidating elements for subsequent filtering. Concurrently, the reasons for potential hypothesis invalidation due to incorrect assumptions are summarized. This deep verification helps Co-Scientist detect subtle errors within complex hypotheses, such as flaws in reasoning or inaccurate experimental protocols. An identified error doesn’t necessarily invalidate the core hypothesis; the Reflection agent assesses whether the incorrect assumption is fundamental to the hypothesis and incorporates this reasoning into the review. Non-fundamental errors can be addressed during subsequent refinement stages. An example deep verification review is provided in Supplementary Note 10.6 for the previously introduced ALS hypothesis. We also show another example of a deep verification review via probing questions in the context of drug repurposing for AML in Supplementary Note 10.7.
- **Observation review.** In addition, the Reflection agent also explores whether a given hypothesis can account for long-tail observations from prior experimental results. This review aims to determine if the hypothesis can provide insights on existing experimental findings and observed phenomena within relevant articles. For each observation, the agent assesses if the hypothesis is a superior explanation over existing ones, assuming its validity. Positive observations are summarized and appended to the hypothesis. Note that this review often completes without any important findings (as in the case of the ALS hypothesis example). An example prompt to generate observations is provided in Supplementary Note 9.2. An illustrative example of an observation review is provided in Supplementary Note 10.8 in the context of an alternate hypothesis for explaining a mechanism of anti-microbial resistance.
- **Simulation review.** The Reflection agent also reviews hypotheses by simulating them in a step-wise fashion (e.g., simulating the mechanism of action or the proposed experiment in the proposal). This simulation allows the agent to identify and summarize potential failure scenarios. This review method is built on the assumption that frontier LLMs may have developed an internal world model of science that enables them to simulate and accurately predict various scientific phenomena.
- **Recurrent/tournament review.** The Reflection agent adapts its full reviews based on Co-Scientist’s growing knowledge. By analyzing reviewed hypotheses and results of the tournament conducted by the Ranking agent, the Reflection agent identifies recurring issues and improvement opportunities, refining its reviews accordingly.

Additionally, Co-Scientist can incorporate reviews from expert scientists to guide ranking and improvements (further discussed in the “Expert-in-the-loop” section). A key aim is to have the Reflection agent generate a comprehensive set of reviews from multiple angles covering the common methods scientists employ when critiquing and refining research hypotheses and proposals.

#### Ranking agent.

Co-Scientist explores numerous hypotheses and research proposals towards a research goal, necessitating a ranking mechanism to prioritize scientists’ time and computational resources toward the most promising candidates. This task is performed by the Ranking agent. The agent orchestrates an Elo-based tournament ^31^ to automatically evaluate and rank all hypotheses, providing supporting rationale. This ranking serves to communicate to scientists an ordered list of research hypotheses and proposals aligned with the research goal. Despite its assumptions and limitations ^32^, Elo remains a good proxy for relative ranking, and it has previously been applied to rank extracted patterns and ideas in games ^33^. In the future, extensions may be considered ^34^. We set the initial Elo rating of 1200 for the newly added hypothesis.

Because the tournament is computationally intensive, the Ranking agent employs several optimization strategies. Top-ranked hypotheses are compared pairwise in tournament matches through multi-turn scientific debates ^35^. This mitigates ordering bias and focuses on novelty, correctness, and testability. Lower-ranked hypotheses undergo single-turn comparisons in a pairwise fashion in their tournament match. The agent concludes each comparison with a decision regarding which hypothesis is better. Supplementary Note 9.3 shows example prompts. Supplementary Note 10.9 shows an example of the Ranking agent conducting a scientific debate match in a tournament to compare two hypotheses.

The Ranking agent prioritizes tournament matches as follows: (1) hypotheses are more likely to be compared with similar ones (based on the Proximity agent’s graph, described in the next section); (2) newer and top-ranking hypotheses are prioritized for participation in tournament matches. Successful hypotheses quickly achieve favorable rankings and this informs the tournament state for subsequent iterations.

#### Proximity agent.

The Proximity agent calculates the similarity between research hypotheses and proposals, and builds a proximity graph, taking into account the specific research goal. Although it doesn’t directly participate in hypothesis generation, the Proximity agent assists the Ranking agent in organizing tournament matches and showcasing a diverse range of ideas related to the research goal. This allows scientists to quickly explore areas of interest and easily identify related concepts.

#### Evolution agent.

The Evolution agent continuously refines and improves existing hypotheses and proposals using several strategies including:

- **Enhancement through grounding.** Here the agent attempts to improve hypotheses by identifying weaknesses, generating search queries, retrieving and reading articles, suggesting improvements and elaborating on details to fill reasoning gaps.
- **Coherence, practicality and feasibility improvements.** The agent aims to address issues and creates more coherent hypotheses, potentially rectifying underlying problems with invalid initial assumptions. The agent also refines the hypotheses to make them more practical and feasible. Supplementary Note 9.4 provides an example of the feasibility improvement prompt.
- **Inspiration from existing hypotheses.** The agent additionally creates new hypotheses inspired by single or multiple top-ranked hypotheses.
- **Combination.** The agent also attempts to directly combine the best aspects of several top-ranking hypotheses to create new hypotheses.
- **Simplification.** The agent simplifies hypotheses for easier verification and testing.
- **Out-of-box thinking.** The agent also explores out-of-the-box ideas by moving away from a subset of hypotheses and generating divergent ones. Supplementary Note 9.4 provides an example prompt for this.

The Evolution agent generates new hypotheses; it doesn’t modify or replace existing ones. This strategy protects the quality of top-ranked hypotheses from flawed improvements, as each new hypothesis must also compete in the tournament. The evolution of research hypotheses and proposals also allows Co-Scientist to iteratively combine different improvement techniques and gradually improve the quality of the results.

#### Meta-review agent.

The Meta-review agent plays a crucial role in Co-Scientist’s feedback loop, enabling self-improvement in scientific thinking and reasoning. This agent operates on the tournament state and summarizes common patterns identified in reviews and scientific debates in the tournament matches into a meta-review critique.

By synthesizing insights from all reviews, the meta-review provides valuable feedback to the Reflection agent, leading to more thorough and reliable future reviews. This helps prevent oversight of critical details. Consider the illustrative example of a identifying a repurposing drug candidate for ALS as a research goal: while only 90% of individual reviews might correctly identify a blood-brain barrier permeability issue in a proposed candidate, the meta-review ensures that all future reviews by the Reflection agent definitively address this crucial factor. Hypothesis and research proposal generation is also enhanced by the meta-review’s identification of recurring issues. While the Generation agent uses this feedback selectively to avoid over-fitting to these review critiques, it helps prevent the recurrence of common issues.

Supplementary Note 9.5 provides an example prompt for the meta-review. In Supplementary Note 10.10, we showcase an example of the summarized meta-review critique generated for the reviews of the previously introduced ALS mechanism hypotheses.

#### Research overview generation.

At the end of the Co-Scientist computation, the Meta-review agent synthesizes top-ranked hypotheses into a research overview, providing a roadmap for future research. This overview outlines potential research areas and directions relevant to the research goal, justifying their importance and suggesting specific experiments within each. Each area includes illustrative example topics. The research overview also serves as an additional input to the Generation agent in subsequent iterations. The research overview serves to effectively map the boundary of current knowledge relevant to the research goal in Co-Scientist and helps highlight future areas of exploration. In Supplementary Note 10.11, we show an example of a research overview for the ALS mechanism research goal. The Meta-review agent can further format these overviews using constrained decoding techniques ^36^ to adhere to common research publication and grant formats (e.g., National Institutes of Health (NIH) Specific Aims Page format). We demonstrate the effectiveness of this in subsequent sections.

#### Research contacts identification.

The Meta-review agent also uses prior literature review to suggest qualified domain experts for research hypotheses and proposal review, including the reasoning behind each suggestion. These potential contacts are summarized in the research overview, providing researchers with additional perspectives and potential avenues for collaborations. An example research contact (with the researcher name redacted) is shown in Supplementary Note 10.12.

#### Expert-in-the-loop interactions with Co-Scientist.

Co-Scientist empowers scientists to actively steer and guide the system through an expert-in-the-loop design (Fig. 1a–b). Scientists can interact with the system in several ways. The typical interaction between Co-Scientist and a human follows a structured process:

- **Research goal definition:** The process begins with a scientist defining the high-level research objective. This involves writing a detailed prompt that can include the specific research question, known constraints of the hypotheses solution space, desired attributes of the output, and relevant background literature and data. With proper goal definition, scientists can direct Co-Scientist to follow up on specific research directions (for example restricted to a smaller collection of prior publications). When this research is referenced in the research goal, Co-Scientist can prioritize generation methods that can access and synthesize it.
- **Goal refinement:** The scientist can refine the initial research goal in light of the generated hypotheses and research overview.
- **Providing review:** The scientist can also provide manual reviews of generated hypotheses, which Co-Scientist uses to evaluate and improve the hypotheses and proposals.
- **Providing ideas and hypotheses:** In the user interface, scientists are allowed to contribute their own hypotheses and proposals for inclusion in the tournament, where they are ranked alongside and can be combined with system-generated hypotheses and proposals.
- **Final review and selection:** After the Co-Scientist run is complete, the scientist is presented with a ranked list of hypotheses and a synthesized research overview from the Meta-review agent. The expert then invests time in reviewing the top-ranked proposals to select the most promising candidates for further experimental validation.

This workflow empowers scientists to guide Co-Scientist at critical junctures. As illustrative examples, we quantified the human time investment for our main validation studies. For the AML drug repurposing study, the initial prompt, defining the goal to find novel combination therapies, required less than one hour of an expert clinician’s time. After the system’s complex run, the final review and selection of promising candidates for *in vitro* testing took roughly three hours. Similarly, the fibrosis target discovery and AMR mechanism generation tasks each required comparable, similar time investments from experts for setup and final review. The scientists and experts featured in our validations have noted that Co-Scientist accomplishes work that would otherwise require days and even weeks of valuable scientists’ time.

#### Tool use in Co-Scientist.

Co-Scientist leverages various tools during the generation, review, and improvement of hypotheses. Web search and retrieval are primary tools, important for grounded, up-to-date hypotheses. For research goals that explore a constrained space of possibilities (e.g., all known cell receptors of a specific type or all FDA-approved drugs), Co-Scientist agents utilize domain-specific tools, such as open databases, to constrain searches and generate hypotheses. Co-Scientist can also index and search a private repository of publications and experimental data specified by the scientist. Finally, the system can utilize and incorporate feedback from specialized AI models like AlphaFold. We demonstrate this qualitatively with a protein design example in the Supplementary Note 11 and Supplementary Fig. 9.

#### Ablation analysis.

To validate the contributions of Co-Scientist’s core components and agents, we performed a series of quantitative ablation studies (details in Supplementary Note 3 and Supplementary Fig. 2–6). These analyses revealed that our architectural choices provide tangible benefits to performance and robustness of the overall system.

Specifically, we quantified the value of our multi-faceted Generation agent, showing that a diverse set of generation strategies contributes to the creation of correct hypotheses across different benchmarks.

Critically, the Reflection agenťs ability to assess novelty was shown to be dependent on its integration with an external search tool. On a dataset of published ideas that should be rated as non-novel, the agent without search incorrectly assigned a high auto-evaluation novelty score of 6.14 (out of 10), while the agent with search correctly assigned a low novelty score of 2.38 (out of 10). The search tool also enhanced review accuracy, raising the average auto-evaluation correctness score from 7.4 to 8.46 (out of 10) for these known-correct ideas and increasing the Area Under the Curve (AUC) on the GPQA benchmark from 0.643 to 0.651 in our run using Gemini 2.0 Flash as the base foundational LLM for the agents in the system.

Furthermore, we demonstrated that using the scientific debate prompt rather than simple comparison prompt for the Ranking agent can significantly enhance ranking accuracy for high-quality hypotheses and, critically, reduces the positional bias.

Our analysis of the Evolution agent confirms that its iterative refinement process is crucial, boosting precision on GPQA from 70.9% to 75.4%, and increasing the average research hypothesis quality score (auto-evaluation) on the constructed dataset from 4.7 to 5.6.

The effectiveness of our Proximity agent was also validated, as its semantic similarity scores showed a strong correlation with the actual quality score differences between hypotheses, ensuring a comprehensive exploration of the problem space.

Finally, we found that Meta-review agent measurably improves the quality of correctness reviews, increasing AUC for predicting a correct solution from 0.521 to 0.597 in our constructed dataset, and 0.629 to 0.634 in the GPQA diamond dataset.

These results provide important quantitative evidence and support that our multi-agent architecture and specialized prompting strategies are critical design choices that lead to more accurate, robust, and reliable scientific hypothesis generation and evaluation.

#### Statistics and reproducibility.

No statistical methods were used to predetermine sample sizes. For computational evaluations, sample sizes (n = 203, n = 15, and n = 11 research goals) were chosen to ensure robust statistical averaging and broad representation across diverse scientific domains. For *in vitro* validations, five distinct AML cell lines were tested in independent biological triplicates (n = 3). This sample size was not predetermined by statistical methods but was chosen based on widely accepted standard practices for preliminary *in vitro* dose-response viability screening. Given the large effect sizes typical of such preliminary pharmacological assays, three independent biological replicates provide the necessary degrees of freedom to calculate standard deviation, assess assay consistency, and robustly fit non-linear regression curves for IC_50_ estimation. All attempts at replication in both computational and *in vitro* were successful. The human expert evaluation of the LLM-generated outputs was explicitly blinded, ensuring that independent domain experts were completely unaware of which model generated the hypotheses they were scoring. Blinding was not applicable to the *in vitro* cell viability screening assays because these experiments involve standardized, automated multimode microplate reader readouts. All cell lines were authenticated by their respective providers.

## Extended Data

**Extended Data Table 1 |:** IC_50_ of the drugs used in the drug combination experiments in AML cell lines, MOLM-13 and KG-1a. The table presents the half-maximal inhibitory concentrations (IC50, in μM) for 11 individual drugs evaluated in two acute myeloid leukemia (AML) cell lines, MOLM-13 and KG-1a. These single-agent efficacies were determined to establish the baseline dose-response behaviors required for the subsequent drug combination synergy analyses. Cell viability was assessed following 72 to 96 hours of drug exposure. IC50 values were estimated using non-linear regression curve fitting. “No response” indicates that the drug failed to achieve 50% growth inhibition within the tested concentration range. All experiments were performed in biologically independent triplicates (n = 3).

Drug	IC_50_ (μM)	
	MOLM-13	KG-1a
MSA-2	No response	No response
Sulfasalazine	No response	No response
CB-839	16.98	8.79
JNJ-64619178 (Onametastat)	0.0046	0.024
Pinometastat	No response	No response
Selinexor	0.039	0.055
JQ1	0.011	0.12
Venetoclax	0.009	0.006
Olaparib	2.12	7.1
SNDX	0.010	No response
Palbociclib	0.026	0.07

**Extended Data Table 2 |:** Summary of the drug combination synergy experiments. The table categorizes the overall in vitro interaction profiles of seven Co-Scientist nominated drug combinations, including doublet and triplet combinations, evaluated in two acute myeloid leukemia (AML) cell lines, MOLM-13 and KG-1a. Pharmacological interactions were quantitatively assessed using the Chou-Talalay combination index (CI) method for doublets, and Highest Single Agent (HSA) and Bliss independence models for triplets. The interaction outcomes are classified into three categories, “Synergistic” (exhibiting CI < 1 or positive excess fractional effect), “Not synergistic” (additive or antagonistic effects), and “Mixed response” (exhibiting context-dependent effects, transitioning between synergy and antagonism depending on the dose or effect level). All experiments were performed in biologically independent triplicates (n = 3).

Drug combination	MOLM-13	KG-1a
JNJ-64619178 + Selinexor	Synergistic	Mixed response
Palbociclib + Selinexor	Synergistic	Synergistic
JNJ-64619178 + SNDX-5613	Synergistic	Mixed response
CB-839 + Sulfasalazine	Not synergistic	Not synergistic
Venetoclax + Pinometostat	Mixed response	Synergistic
JNJ-64619178 + Selinexor + SNDX-5613	Synergistic	Synergistic
JQ1 + Olaparib + MSA-2	Synergistic	Not synergistic

## Supplementary Material

Supplement 1

## Figures and Tables

**Table 1 |:** Three real-world applications in biomedicine for end-to-end validation of Co-Scientist. The table summarizes three scientific tasks selected to evaluate the hypothesis generation capabilities of Co-Scientist. The chosen applications span varying biological disciplines and are categorized by four axes, inherent challenge (the primary scientific objective), complexity (the depth of reasoning required), scale (data availability and experimental feasibility), and unknown elements (the boundaries of the hypothesis search space). These progressively demanding tasks illustrate Co-Scientist’s generalizability and its capacity to navigate both constrained problem spaces and open-ended explorations.

Application	Drug repurposing	Novel treatment target discovery	Explain mechanism of gene transfer evolution
Challenge	Complex search	Identifying novel targets	Understanding complex systems
Complexity	Medium	High	Very high
Scale	Moderate, data-limited	Moderate, experiment-limited	Large, data and computation-limited
Unknown elements	Constrained	Large	Vast and dynamic

## Data Availability

Except for the three real-world validation tasks (drug repurposing for AML, novel target discovery for liver fibrosis, mechanism explanation of gene transfer evolution), the remaining datasets used for development, benchmarking and evaluation of the systems are open source or otherwise accessible publicly with permissions. Specifically, the GPQA diamond dataset is publicly available at Hugging Face (https://huggingface.co/datasets/Idavidrein/gpqa). The Cancer Dependency Map (DepMap) Q2 2024 data used for computational sanity checks is publicly available at the DepMap portal (https://depmap.org/portal/). The curated drug targets dataset from the Open Targets Platform is available at https://platform.opentargets.org/downloads.

## Code availability

The full source code for the Co-Scientist system is not publicly available. Due to the deep integration of the Co-Scientist multi-agent framework with proprietary internal infrastructure, the immense computational resources required for massive test-time scaling, and the safety implications of unmonitored autonomous agentic use of such capable AI systems, we are unable to publicly release the full source code or provide broad access immediately.

Instead, to enable and accelerate research on important scientific problems, we are initiating an experimental access program. We request scientists interested in solving important scientific problems to reach out and we will provision access subject to computational resources. In due course, we expect to provide broader access to thousands of scientists across the scientific community via Google APIs and bespoke interfaces over time. To further aid transparency and reproducibility, we have provided comprehensive pseudocode detailing the multi-agent orchestration and tournament logic (Supplementary Note 8), alongside the exact system prompts utilized (Supplementary Note 9).

The foundational LLM model, Gemini, used in Co-Scientist is publicly available via APIs, Google AI studio, the Gemini App and other surfaces. Further, important technical details on Gemini have been described extensively in corresponding technical reports ^8,14,37^.

The system, algorithms, and analysis scripts were developed using Python (version 3.11.7). Data handling and visualization were performed using pandas (version 2.1.4), numpy (version 1.26.4), seaborn (version 0.12.2), and matplotlib (version 3.8.0). For *in vitro* validations, dose-response curve fitting and IC_50_ estimation were performed using GraphPad Prism (version 10.6.0, GraphPad Software). Dose-effect data from single-agent and combination treatments were analysed using Julius AI statistical software (accessed November 2025).
